# The risk of melanoma with rasagiline compared with other antiparkinsonian medications: A retrospective cohort study in the United States medicare database

**DOI:** 10.1002/pds.5422

**Published:** 2022-04-06

**Authors:** Catherine B. Johannes, Catherine W. Saltus, James A. Kaye, Brian Calingaert, Sigal Kaplan, Mark Forrest Gordon, Elizabeth B. Andrews

**Affiliations:** ^1^ Department of Epidemiology RTI Health Solutions Waltham Massachusetts USA; ^2^ Department of Epidemiology RTI Health Solutions Research Triangle Park North Carolina USA; ^3^ Global Patient Safety & Pharmacovigilance Teva Pharmaceutical Industries Ltd. Netanya Israel; ^4^ Global Specialty R&D Teva Branded Pharmaceutical Products R&D, Inc. West Chester Pennsylvania USA

**Keywords:** antiparkinsonian drugs, Medicare, melanoma, Parkinson's disease, rasagiline

## Abstract

**Purpose:**

Compare the risk of melanoma between initiators of rasagiline or other antiparkinsonian drugs (APDs) in a Parkinson's disease (PD) population.

**Methods:**

A retrospective cohort study was conducted in the US Medicare claims research database (2006–2015) in adults aged ≥65 years with PD claims. Other APD initiators were randomly matched (4:1) to rasagiline initiators on age, sex, and cohort entry year. Cutaneous melanoma events were identified by a validated claims algorithm. Incidence rates (IRs), incidence rate ratios (IRRs), and Cox‐adjusted hazard ratios (HRs) for melanoma comparing rasagiline with other APD initiators were calculated and analyzed by duration of study medication use and cumulative dose of rasagiline. Potential indicators of surveillance bias were explored.

**Results:**

Among 23 708 rasagiline initiators and 96 552 matched APD initiators, the crude IR of melanoma/100 000 person‐years was 334.3 (95% confidence interval [CI], 291.5–381.6) and 208.2 (95% CI, 190.1–227.5), respectively (crude IRR 1.61; 95% CI, 1.36–1.89). The adjusted HR was 1.37 (95% CI, 1.14–1.65) and increased with longer rasagiline exposure and higher cumulative rasagiline doses. Rasagiline initiators more frequently had dermatologist visits or skin biopsies before cohort entry than APD initiators and had a higher incidence of nonmelanoma skin cancer during follow‐up (crude IRR, 1.44; 95% CI, 1.35–1.54).

**Conclusions:**

A small increased incidence of melanoma with exposure to rasagiline compared with other APDs was observed. Although the pattern with dose and duration is consistent with a hypothesized biologic effect, the increased skin cancer surveillance among rasagiline users suggests surveillance bias as a contributing explanation for the observed results.

**Key Points**

- The risk of melanoma in new users of rasagiline compared with new users of other antiparkinsonian medications is unknown.
- In this retrospective, matched cohort study conducted in 23 708 rasagiline initiators and 96 552 initiators of other antiparkinsonian medications in the US Medicare database, the risk of melanoma was 37% higher with rasagiline exposure than with exposure to other antiparkinsonian medications and increased with higher dose and longer duration of use.
- The observed effect was weakly positive, and there was also evidence of increased skin cancer surveillance among rasagiline new users that suggests surveillance bias as a contributing explanation.

## 1 INTRODUCTION

Parkinson's disease (PD) is a degenerative disorder of the central nervous system characterized by bradykinesia, rest tremor, rigidity, and impaired postural reflexes.^1^ The onset of PD is uncommon before the age of 50 years, and the incidence and prevalence increases sharply after age 60 years.^2^, ^3^ Symptomatic treatment of motor symptoms of PD is tailored according to disease stage and modified over time as the disease progresses.^4^ For patients with mild motor symptoms, a monoamine oxidase type B (MAO‐B) inhibitor may be initiated, while a dopamine agonist or levodopa may be initiated for more severe symptoms.^5^

There is epidemiologic evidence that patients with PD have an increased risk of developing melanoma and possibly other skin cancers, whereas they face a decreased risk of most other cancers.^6^, ^7^, ^8^, ^9^, ^10^, ^11^, ^12^, ^13^, ^14^ This increased risk for melanoma has been estimated at about 2‐fold in a recent meta‐analysis.^14^ The possible mechanisms for the association between PD and melanoma are not known, but appear to be related more to factors associated with the PD itself rather than dopaminergic therapy for PD.^6^, ^7^, ^15^, ^16^, ^17^ For example, there are a number of genes and proteins in which mutations or other alterations are common to both PD and melanoma, including factors that contribute to melanin biosynthesis, cellular detoxification, oxidative stress response, and cellular trafficking pathways that could link the two diseases.^7^ Risk of melanoma related to other PD therapies has not been well studied.

Rasagiline is a selective, irreversible MAO‐B inhibitor indicated for the treatment of PD, with approval in Europe in 2005 and in the United States in 2006. During the rasagiline development program, several cases of melanoma were diagnosed, prompting an inquiry into the cause of these events and leading to increased clinical monitoring of patients and a request from the US Food and Drug Administration to conduct a postmarketing study.^17^ The primary objective of this study was to compare the risk of incident melanoma between rasagiline new users and new users of other antiparkinsonian drugs (APDs). To explore potential surveillance bias (i.e., increased detection of skin neoplasms due to possible increased monitoring among new users of rasagiline than among users of other APDs), the incidence rates (IR) of nonmelanoma skin cancer (NMSC) were estimated, and health resource use was described.

## 2 METHODS

### 2.1 Study design

This retrospective cohort study with a new‐user, active comparator design^18^ used data from the US Medicare research claims database from 1999 through 2015. Medicare, a federally sponsored health insurance program for adults aged ≥65 years or with a permanent disability, consists of Part A, hospital insurance; Part B, physician services and outpatient care; and Part D, outpatient prescription drug coverage (available since 2006). Medicare beneficiaries include more than 98% of the US population aged ≥65 years,^19^ about two‐thirds of whom are fee‐for‐service enrollees and are captured in the Medicare database.

The Research Triangle Institute institutional review board approved the study. The Centers for Medicare and Medicaid Services (CMS) Privacy Board approved access to Medicare research identifiable files through the Virtual Research Data Center (VRDC).

### 2.2 Study population

The study population comprised Medicare fee‐for‐service enrollees aged ≥65 years with at least 6 months of continuous enrollment in Medicare Parts A, B, and D and at least two physician or outpatient claims or at least two inpatient claims with an International Classification of Diseases, Ninth Revision, Clinical Modification (ICD‐9‐CM) code of 332.0 (paralysis agitans) or a Tenth Revision, Clinical Modification (ICD‐10‐CM) code of G20 (PD) on or before the cohort entry date. All available lookback time in the Medicare claims data, depending on the enrollment date of each beneficiary, was used to assess PD. The cohort entry date was the date of the first dispensed prescription for a new exposure of interest during the study period (2006–2015) in patients with no record of a previous dispensing of that medication. Excluded, using all available lookback time, were patients with codes for secondary parkinsonism: neuroleptic‐induced parkinsonism or parkinsonism due to drugs (ICD‐9‐CM code of 332.1 or ICD‐10‐CM code of G21.11, G21.19, or G21.8) or a history or melanoma on or before cohort entry. Patients were also excluded if they did not reside in a US state or the District of Columbia, were entitled to Medicare because of end‐stage renal disease, or had a history of melanoma on or before the cohort entry date.

### 2.3 Exposures

Exposures were evaluated from claims for dispensed prescriptions for rasagiline (primary exposure) or any other APD approved in the United States (active comparator exposure) (eTable A‐1, Supplement). The first study exposure newly initiated during the study period was the index medication. Two main exposure cohorts were created: (1) all new users of rasagiline (rasagiline cohort) and (2) a random sample of new users of any non‐rasagiline APD (APD cohort). New users of rasagiline were included in the rasagiline cohort regardless of whether they were current or previous users of another APD. New users of any non‐rasagiline APD drug were included in the APD cohort regardless of whether they were current or previous users of any other APD except rasagiline. Follow‐up time for patients in the APD cohort who subsequently initiated rasagiline treatment was censored at the date of the first dispensing for rasagiline; if all entry criteria for the rasagiline cohort were met at the time of switching, the remaining follow‐up time was counted in the rasagiline cohort.

The APD cohort was matched to the rasagiline cohort in a 4:1 ratio based on year of age, sex, and calendar year of cohort entry. For the primary analysis reported in this article, patients using selegiline, a MAO‐B inhibitor in the same class as rasagiline, before or on the cohort entry date were excluded so that the effect of rasagiline could be studied apart from a possible class effect. Selegiline users were included in sensitivity analyses.

Person‐time at risk for each cohort was categorized by the index medication use as current use—from the prescription filling date to the last day of supply, with subsequent prescriptions separated by ≤30 days counted as continuous current use; recent use—from the end of current use to the earlier of 365 days later or a new dispensing of the index drug (thus resuming current use); and past use—from the end of recent use to the end of follow‐up or a new dispensing of the index drug.

In a secondary study, two additional cohorts were evaluated—patients with PD with incident or prevalent non‐rasagiline APD treatment, and patients without any record of PD or APD treatment —to evaluate the incidence of melanoma in patients with and without PD (eMethods 1.1, eTables B‐1 to B‐3, Supplement).

### 2.4 Outcomes

The primary outcome was the first occurrence of cutaneous melanoma after cohort entry. A claims algorithm of ICD‐9‐CM diagnosis code 172.XX or ICD‐10‐CM code C43.XX on at least two outpatient or physician visit claims on different dates or on at least one hospital claim was used to identify potential melanoma cases. These codes exclude melanomas arising in skin of genital organs and sites other than skin. Abstracted medical records of potential cases were reviewed to verify the diagnosis, identify melanoma stage, and calculate the positive predictive value (PPV) of the case‐finding algorithm (eMethods 1.2 and eTable B‐4, Supplement).

The secondary outcome, NMSC, was ascertained by using a validated published algorithm developed using payer claims data from a US health system.^20^ A random sample of 100 potential NMSC cases was validated by medical record review (eMethods 1.2 and eTable B‐5, Supplement).

### 2.5 Study follow‐up

Follow‐up began on the cohort entry date and ended on the earliest date of the following: first claim for melanoma; first claim for selegiline; disenrollment from Medicare Part A, B, or D; switch to a managed care plan; death; or end of study period (December 31, 2015).

### 2.6 Potential confounding variables

Potential confounding factors were age, sex, race/ethnicity, region of residence, Medicare Part D low‐income subsidy status (proxy for socioeconomic status), the Charlson Comorbidity Index (CCI),^21^, ^22^ individual medical conditions that comprise the CCI, asthma, organ transplant, immunosuppressive disorders, hyperlipidemia, and comedications (eTable A‐2, Supplement).

### 2.7 Statistical analysis

Analyses were performed using SAS Enterprise Guide within the CMS VRDC.

Descriptive analyses compared baseline characteristics between the two exposure cohorts. Incidence rates and 95% confidence intervals (CIs) for melanoma and NMSC were calculated by dividing the number of events detected by the algorithm by the total person‐time.^23^ Exposure category (overall, current, recent, and past), duration of index medication exposure, and cumulative‐dose of rasagiline were examined.

Crude and Mantel–Haenszel–adjusted incidence rate ratios (IRRs) and 95% CIs were estimated. Adjusted hazard ratios (HRs) and 95% CIs of melanoma were calculated comparing the rasagiline cohort with the APD cohort by using Cox regression models with time‐varying covariates.

### 2.8 Surveillance bias analysis

To evaluate possibly increased surveillance for skin neoplasms in rasagiline initiators than in other APD initiators, we described the following for each cohort: (1) frequency of health care resource use, including dermatology visits and skin biopsies, in the 180 days before and after the cohort entry date; (2) melanoma stage at diagnosis; (3) melanoma incidence rates during the 6 months after cohort entry; and (4) incidence rates of NMSC.

### 2.9 Additional analyses

A post‐hoc quantitative bias analysis^24^ was performed to determine how strong an unmeasured confounder would have to be to account for the observed effect estimates (eMethods 1.3, Supplement). A sensitivity analysis was conducted to assess the impact of excluding selegiline users from the cohorts and truncating follow‐up time at the first use of selegiline (detailed results not reported). A post hoc sensitivity analysis was performed to examine the effect on melanoma incidence rate estimates in both study cohorts by limiting the cohorts to patients with at least 12 and 24 months of precohort entry date enrollment in Medicare parts A and B instead of 6 months.

## 3 RESULTS

### 3.1 Participants and baseline characteristics

A total of 23 708 and 96 552 patients were included in the rasagiline and matched APD cohorts (see flow diagram for study attrition in the Supplement (eFigure A‐1). A similar percentage of patients in the rasagiline and APD cohorts, respectively, were male (55.1% vs. 54.5%) and aged 85 years and older (10.8% vs. 10.9%), and a slightly higher percentage were white (89.2% vs. 87.2%) (Table 1). A lower percentage of patients in the rasagiline cohort than the APD cohort had a Part D low‐income subsidy status (23.4% vs. 38.7%). Patients in the APD cohort had a higher prevalence of most individual comorbidities, including cardiovascular and cerebrovascular disease, chronic pulmonary disease, diabetes, and dementia, and 39.4% had a CCI score ≥5 compared with 25.0% in the rasagiline cohort. Non‐rasagiline comedications were distributed similarly between the two cohorts.

**TABLE 1:** Baseline characteristics of patients in each cohort

Characteristic	Rasagiline cohort (*N* = 23 708),^c^ *n* (%)	APD cohort (*N* = 96 552),^c^ *n* (%)
Sex		
Female	10 635 (44.9)	43 894 (45.5)
Male	13 073 (55.1)	52 658 (54.5)
Age, years		
65–69	5045 (21.3)	19 740 (20.4)
70–74	5961 (25.1)	24 308 (25.2)
75–79	5819 (24.5)	24 017 (24.9)
80–84	4314 (18.2)	17 915 (18.6)
85+	2569 (10.8)	10 572 (10.9)
Calendar year of cohort entry		
2006	1402 (5.9)	5821 (6.0)
2007	1929 (8.1)	8092 (8.4)
2008	1722 (7.3)	7178 (7.4)
2009	1992 (8.4)	8572 (8.9)
2010	2387 (10.1)	9770 (10.1)
2011	2581 (10.9)	10 518 (10.9)
2012	2661 (11.2)	10 632 (11.0)
2013	3113 (13.1)	12 389 (12.8)
2014	3503 (14.8)	13 916 (14.4)
2015	2418 (10.2)	9664 (10.0)
Race/ethnicity		
White	21 151 (89.2)	84 212 (87.2)
Black	725 (3.1)	5313 (5.5)
Asian	649 (2.7)	2294 (2.4)
Hispanic	567 (2.4)	2587 (2.7)
Other	492 (2.1)	1809 (1.9)
Unknown	124 (0.5)	337 (0.3)
Geographic region of residence		
Midwest	4651 (19.6)	25 169 (26.1)
Northeast	4653 (19.6)	16 656 (17.3)
South	9147 (38.6)	38 419 (39.8)
West	5257 (22.2)	16 308 (16.9)
Low‐income subsidy status		
Yes	5558 (23.4)	37 355 (38.7)
No	18 150 (76.6)	59 197 (61.3)
Comorbidities		
CCI comorbidity score^a^		
0	4828 (20.4)	12 020 (12.4)
1–2	7594 (32)	24 087 (24.9)
3–4	5365 (22.6)	22 393 (23.2)
5 or more	5921 (25)	38 052 (39.4)
History of individual CCI conditions^a^		
Myocardial infarction	2542 (10.7)	15 529 (16.1)
Congestive heart failure	4835 (20.4)	29 622 (30.7)
Peripheral vascular disease	6952 (29.3)	37 819 (39.2)
Cerebrovascular disease	9369 (39.5)	47 738 (49.4)
Dementia	4476 (18.9)	32 300 (33.5)
Chronic pulmonary disease	7296 (30.8)	41 304 (42.8)
Connective tissue disease	1658 (7.0)	8199 (8.5)
Ulcer disease	1410 (5.9)	8562 (8.9)
Mild liver disease	238 (1.0)	1467 (1.5)
Diabetes	4597 (19.4)	21 528 (22.3)
Hemiplegia	957 (4.0)	6316 (6.5)
Moderate or severe renal disease	3284 (13.9)	20 833 (21.6)
Diabetes with end organ damage	3381 (14.3)	21 252 (22.0)
Any malignancy	4980 (21.0)	20 612 (21.3)
Moderate or severe liver disease	88 (0.4)	897 (0.9)
Metastatic solid tumor	503 (2.1)	2560 (2.7)
AIDS	22 (0.1)	164 (0.2)
History of other medical conditions^a^		
Asthma	2641 (11.1)	14 881 (15.4)
Organ transplant	65 (0.3)	385 (0.4)
Immunosuppressive disorders (other than those listed in the CCI)	91 (0.4)	376 (0.4)
Hyperlipidemia	19 334 (81.6)	79 469 (82.3)
Non‐antiparkinsonian comedications^b^		
Antihypertensives/diuretics	12 651 (53.4)	57 025 (59.1)
Antirheumatic agents (other than NSAIDs or corticosteroids)	172 (0.7)	876 (0.9)
Immunosuppressants including corticosteroids	2226 (9.4)	10 886 (11.3)
NSAIDs	3297 (13.9)	14 163 (14.7)

Abbreviations: APD, antiparkinsonian drug; CCI, Charlson comorbidity index; NSAID, nonsteroidal anti‐inflammatory drug.

^a^ Medical conditions were assessed using all available history before or on the cohort entry date.

^b^ Medications were assessed in the 180 days before or on the cohort entry date.

^c^ Cohort sizes are not in an exact 4:1 ratio because selegiline users were removed from both cohorts after matching. This also results in the matching variables (age, sex, and year of cohort entry) not having exactly the same distribution in both cohorts.

The most common index medication in the APD cohort was carbidopa/levodopa (45.2%), followed by ropinirole (12.4%), and pramipexole (9.9%) (eTable A‐3, Supplement). The majority of rasagiline initiators (71.2%) used levodopa with or without carbidopa or a catechol‐*O*‐methyltransferase (COMT) inhibitor in the 180 days before cohort entry, 27.0% used a dopamine agonist, and 6.1% used an anticholinergic medication. Just over half of the rasagiline cohort (51.3%) had no concurrent use of another antiparkinsonian medication at cohort entry, and 32.7% had concurrent use of a single levodopa drug.

### 3.2 Main results

The case‐finding algorithm identified 219 incident melanoma cases in the rasagiline cohort, for an overall incidence rate of 334/100 000 person‐years (95% CI, 292–382), and 486 in the APD cohort, for an overall incidence rate of 208/100 000 person‐years (95% CI, 190–228) (Table 2). The PPV of the algorithm was 82.9% (95% CI, 70.9%–86.4%) (eTable B‐6, Supplement). The crude incidence rate in both cohorts was highest for recent exposure and lowest for past exposure. In the rasagiline cohort, the incidence rate increased with longer duration of exposure, but not in the APD cohort (Table 2). In the rasagiline cohort, the incidence rate tended to increase with higher cumulative doses.

**TABLE 2:** Melanoma incidence by exposure categories, crude incidence rate ratios and adjusted hazards ratios comparing rasagiline initiators with initiators of other antiparkinsonian drugs

Type of exposure during study period	No. of melanoma events	No. of patients	Person‐time, year	Crude incidence rate per 100 000 Person‐years (95% CI)	Crude^a^ IRR (95% CI)	Cox‐adjusted^b^ HR (95% CI)
Rasagiline						
No exposure to rasagiline (APD cohort)^c^	486	96 552	233 440	208.2 (190.1–227.5)	Reference	Reference
Any exposure during study period	219	23 708	65 512	334.3 (291.5–381.6)	1.61 (1.36–1.89)	1.37 (1.14–1.65)
Exposure category^d^						
Rasagiline cohort						
Current	96	23 708	29 179	329.0 (266.5–401.8)	1.58 (1.26–1.97)	1.31 (1.03–1.67)
Recent	69	19 746	16 461	419.2 (326.1–530.5)	2.01 (1.54–2.60)	1.70 (1.30–2.24)
Past	54	9749	19 872	271.7 (204.1–354.6)	1.31 (0.97–1.73)	1.17 (0.86–1.59)
APD cohort						
Current	255	96 552	122 546	208.1 (183.3–235.3)	NA	NA
Recent	128	75 406	54 490	234.9 (196.0–279.3)	NA	NA
Past	103	28 617	56 404	182.6 (149.1–221.5)	NA	NA
Duration of exposure, days						
Rasagiline cohort						
<60	38	23 708	14 563	260.9 (184.7–358.2)	1.25 (0.88–1.75)	1.09 (0.77–1.54)
60–174	54	18 900	16 439	328.5 (246.8–428.6)	1.58 (1.17–2.09)	1.31 (0.97–1.78)
175–500	63	13 103	17 177	366.8 (281.8–469.3)	1.76 (1.33–2.29)	1.51 (1.14–2.01)
>500	64	7157	17 333	369.2 (284.4–471.5)	1.77 (1.34–2.31)	1.55 (1.16–2.06)
APD cohort						
<60	125	96 552	55 180	226.5 (188.6–269.9)	NA	NA
60–174	105	75 531	52 188	201.2 (164.6–243.6)	NA	NA
175–500	120	54 824	59 547	201.5 (167.1–241.0)	NA	NA
>500	136	30 974	66 524	204.4 (171.5–241.8)	NA	NA
Cumulative dose of rasagiline, mg						
<45	38	23 691	14 713	258.3 (182.8–354.5)	1.24 (0.87–1.73)	1.10 (0.78–1.56)
45–<150	54	18 620	17 145	315.0 (236.6–411.0)	1.51 (1.12–2.01)	1.26 (0.94–1.70)
150–420	62	12 510	16 172	383.4 (293.9–491.5)	1.84 (1.39–2.40)	1.57 (1.18–2.08)
>420	65	7028	17 483	371.8 (286.9–473.9)	1.79 (1.36–2.32)	1.54 (1.16–2.06)

Abbreviations: APD, antiparkinsonian drug; CI, confidence interval; HR, hazard ratio; IRR, incidence rate ratio; NA, not applicable.

^a^ The APD cohort was matched to rasagiline cohort on age, sex, and calendar year of cohort entry.

^b^ Adjusted for age (5‐year categories); sex; race; low‐income subsidy status; health care resource use in the 180 days on or before cohort entry (primary care visits, dermatologist visits, neurologist visits, hospitalizations for any cause, hospitalizations for Parkinson's disease, skin biopsies); ever exposure to levodopa, dopamine agonists, catechol‐*O*‐methyltransferase (COMT) inhibitors, or anticholinergics and other antiparkinsonian agents (time‐varying); and comorbidity index score. The results from each of the four sections of the table (any exposure, exposure category, duration of exposure, and cumulative dose) come from separate regression models, each with no exposure to rasagiline as the reference.

^c^ The “no exposure to rasagiline (APD cohort)” is used as the reference group for all crude IRRs and Cox‐adjusted HRs in the exposure categories.

^d^ Categories are in relation to rasagiline for the rasagiline cohort and to the index APDs for the APD cohort. Current exposure is the time from the date of the index prescription dispensing to the last day of supply; recent exposure is from the end of current exposure to the earlier of 365 days later or a new dispensing of the index drug (thus resuming current use); past exposure is from the end of recent use to the earlier of end of follow‐up or a new dispensing of the index drug.

The overall crude IRR of melanoma comparing any rasagiline exposure during the study period with any APD exposure was 1.61 (95% CI, 1.36–1.89) (Table 2). The corresponding crude incidence rate difference was 126 cases per 100 000 person‐years (95% CI, 78–174).

Compared with no exposure, the adjusted HR of algorithm‐defined melanoma for any exposure to rasagiline during the study period was 1.37 (95% CI, 1.14–1.65) (Table 2). Results were similar when restricting the analysis to the subset of medical record–confirmed melanoma cases (HR, 1.46; 95% CI, 1.11–1.93). The HR of melanoma was about 50% higher in rasagiline new users with ≥175 days of exposure compared with no exposure and was about 55% higher with cumulative exposure to 150 mg or more of rasagiline than with no exposure (Table 2).

Demographic variables that were independent predictors of melanoma status in the Cox multiple regression model were male sex (HR, 2.13; 95% CI, 1.80–2.53), white race (HR, 2.85; 95% CI, 1.85–4.40), and Part D low‐income subsidy status (HR, 0.56; 95% CI, 0.45–0.69), that is, higher socioeconomic status by this indicator was positively related to melanoma risk. Other predictors of melanoma risk among health utilization variables assessed in the 180 days before the cohort entry date compared with none were ≥2 dermatologist visits (HR, 1.89; 95% CI, 1.43–2.50), ≥2 skin biopsies (HR, 2.34; 95% CI, 1.60–3.41), ≥2 hospitalizations for PD (HR, 2.25; 95% CI, 0.55–9.21), and ≥2 hospitalizations for any cause (HR, 0.66; 95% CI, 0.49–0.87). Medication variables associated with melanoma risk were ever use of a dopamine agonist (HR, 1.18; 95% CI, 1.01–1.38) and ever use of a COMT inhibitor (HR, 0.76; 95% CI, 0.58–0.99) compared with no use.

### 3.3 Surveillance bias analysis

Rasagiline users were more likely than the APD cohort to have had ≥1 dermatologist visits or ≥1 skin biopsies (potential indicators of increased surveillance for skin cancers) during the 180 days before or on the cohort entry date. (Table 3); similar results were observed in the 180 days after cohort entry for those with at least 180 days of follow‐up. However, other types of health care contact that are less specifically related to the detection of skin cancers, including primary care visits, hospitalizations for any cause, and hospitalizations for PD were more prevalent in the APD cohort than in the rasagiline cohort.

**TABLE 3:** Evaluation of surveillance bias, descriptive results

Variable	Rasagiline cohort (*N* = 23 708)	APD cohort (*N* = 96 552)
Health care use in the 180 days before and including the cohort entry date, %		
≥3 primary care physician visits	47.4	54.9
≥2 neurologist/neurosurgeon visits	55.7	44.4
≥2 hospitalizations for any cause	8.1	21.6
≥1 hospitalizations for Parkinson's disease	1.3	2.1
≥1 dermatologist visits	15.0	10.5
≥1 skin biopsies	8.1	5.7
Incidence of nonmelanoma skin cancer per 100 000 person‐years	2810	1945
Stage of melanoma among confirmed cases		
No. of confirmed cases with staging information	122	227
Early stage, %^a^	63.1	57.7

*Note*: The number of confirmed cases in each cohort is the denominator for calculating the stage percentages. Late stage could not be reported because, according to Medicare privacy rules, any cell with a count of 1–10, or any cell that allows a count of 1–10 to be derived from other reported cells or information, cannot be reported.

Abbreviation: APD, antiparkinsonian drug.

^a^ Early stage comprises stages 0 and 1A.

Among confirmed cases of melanoma for which staging information at diagnosis was available in the medical records, a slightly higher percentage in the rasagiline cohort (63.1%) than in the APD cohort (57.7%) were early stage melanomas.

The incidence of melanoma in the 6‐month period after cohort entry among rasagiline new users was 409/100 000 person‐years (95% CI, 300–546), higher than that for new users of other APDs, 224/100 000 person‐years (95% CI, 183–273), during the same period. The crude IRR comparing rasagiline with other APDs in the first 6 months of follow‐up was 1.82 (95% CI, 1.26–2.61), somewhat higher than the IRR for all follow‐up time excluding the first 6 months (1.56, 95% CI, 1.30–1.87). The crude overall incidence rate/100 000 person‐years of NMSC was based on cases defined by the algorithm with a PPV of 83.0% (95% CI, 74.2%–89.8%) (eTable B‐6, Supplement). The incidence of NMSC was higher in the rasagiline cohort (2810; 95% CI, 2656–2970) than in the APD cohort (1945; 95% CI, 1881–2012), with an unadjusted IRR of 1.44 (95% CI, 1.35–1.54).

### 3.4 Additional analyses

#### 3.4.1 Quantitative bias analysis

In the quantitative bias analysis (eTable B‐7, Supplement), we found that a hypothetical confounder with an RR of 4.0 for melanoma and a prevalence of 50% in the rasagiline cohort would require a prevalence of 26% or less in the APD cohort to account for the observed effect.

#### 3.4.2 Analysis of patients with and without PD

A higher incidence of melanoma was found in patients without PD than in those with PD who were prevalent or new users of non‐rasagiline APD (adjusted HR 1.72; 95% CI, 1.31–2.22) (eMethods 1.1 and eTable B‐3, Supplement).

#### 3.4.3 Sensitivity analysis

Extending the precohort entry date minimum enrollment requirement in Medicare parts A and B to at least 12 and 24 months retained 96% and 84% of the study population, respectively. The original crude incidence rate for the rasagiline cohort with 6 months of continuous enrollment (334/100 000 person‐years) changed to 331/100 000 person‐years with a 12‐month minimum enrollment requirement and to 335/100 000 person‐years with a 24‐month minimum requirement. For the matched APD cohort with 6 months of enrollment, the rate was 208/100 000 person‐years, which changed slightly to 210/100 000 and 212/100 000 person‐years, respectively, when minimum requirements of 12 months and 24 months were applied.

In another sensitivity analysis, selegiline users were included. Results were similar to the primary analysis, although the number of selegiline users was small (1839 in the rasagiline cohort and 4556 in the APD cohort).

## 4 DISCUSSION

In this longitudinal study using real‐word data, we observed a 37% increased risk of melanoma in new users of rasagiline relative to new users of non‐rasagiline APDs. This risk increased with higher cumulative doses and longer durations of exposure to rasagiline, a pattern consistent with a hypothesized biologic effect. However, the magnitude of the observed rasagiline effect is small. The results are based on melanoma events detected by a validated claims algorithm with a high PPV (83%), indicating that most algorithm‐detected events were true cases.

A possible contributing explanation for an increased incidence of melanoma in rasagiline users could be surveillance bias, also known as detection bias.^25^ In our study, rasagiline new users were about 40% more likely than new users of another APD to have dermatologist visits and skin biopsies in the 180 days before and after the cohort entry date. A previous study using Medicare claims data (1986–2001) showed that increased skin biopsy rates were associated with a higher incidence of melanoma, particularly early stage melanoma,^26^ and a study in health care professionals (1990–2012) demonstrated a positive association between various health care screening practices and NMSC and, to a lesser extent, melanoma.^27^ These studies provide evidence for surveillance bias in studies of skin neoplasms. Our results may stem from the recommendation in the rasagiline label that was in force during the study period^28^ for patients and providers to monitor for melanomas frequently and on a regular basis.

The IRR for melanoma was higher during the first 6 months after cohort entry, when biological effects of a medication are less plausible, than during the subsequent follow‐up time. This finding adds support to the possible influence of surveillance bias on the association between rasagiline use and melanoma in this study. The observed higher incidence of NMSC in the rasagiline cohort compared with the APD cohort is further evidence for surveillance bias, as an association between NMSC and rasagiline exposure is not expected. The overall unadjusted IRR for NMSC in the rasagiline cohort compared with the APD cohort was 1.44, about the same order of magnitude as the adjusted HR for cases of melanoma (1.37). The slightly higher proportion of early stage melanoma cases in the rasagiline cohort versus the APD cohort is also consistent with surveillance bias. On the other hand, surveillance bias cannot account for the observed pattern of increasing risk with rasagiline dose and duration.

There is considerable evidence in the literature of a positive association between PD and melanoma,^14^ and MAO‐B inhibitors may be prescribed to patients at early stages of PD,^5^ so patients with more advanced stages of PD could be less likely to receive rasagiline. It is possible that patients in the APD cohort whose index therapy was most often a combination of carbidopa and levodopa, were, on average, at later stages of PD than patients in the rasagiline cohort. If so, these patients may have been more likely to have already developed melanoma and therefore be excluded from the cohort, making them appear at a lower risk of melanoma than patients in the rasagiline cohort. Information on PD onset date and stage of PD progression were not available. It is also possible that a melanoma diagnosed after a patient started rasagiline could have originated during prior exposure to another APD.

As this was not a randomized study, an important limitation is the potential for unmeasured confounding from known melanoma risk factors that are not available in the database. Although multivariable adjustment of the imbalance between the exposure cohorts was used, there could still be residual confounding related to unmeasured melanoma risk factors, including genetic abnormalities, family history, history of exposure to ultraviolet radiation, smoking, obesity, education, and income if they were also related to choice of PD therapy.^29^, ^30^ Furthermore, residual confounding from variables that incompletely capture the underlying construct is also possible. Results from the post hoc quantitative bias analysis showed it is unlikely that a single unmeasured confounder would produce an effect strong enough to entirely account for the observed results. However, the combined effect of multiple unmeasured confounders is unknown.

In an analysis of two additional cohorts, a higher incidence of melanoma was found in patients without PD than in those with PD who were prevalent or new users of non‐rasagiline APD. This result differs from findings in other epidemiological studies that have established an association between PD and melanoma.^7^, ^8^, ^14^ However, there were important features of the present study design, including the use of a prevalent PD population and the exclusion of subjects with a history of melanoma, that limit comparison with previous population‐based studies of this association and warrant further investigation.

## 5 CONCLUSIONS

The present study showed a small increased incidence of melanoma among Medicare beneficiaries with PD who initiated therapy with rasagiline compared with age‐ and sex‐matched beneficiaries who initiated a non‐rasagiline APD after adjustments were made for multiple possible confounding factors. Although the pattern with dose and duration is consistent with a hypothesized biologic effect, the association is weak and the observation of increased skin cancer surveillance among new users of rasagiline suggests surveillance bias as a contributing explanation for the observed results. Residual confounding due to incompletely adjusted or unmeasured risk factors could also be present.

## CONFLICT OF INTEREST

Catherine B. Johannes, Catherine W. Saltus, James A. Kaye, Brian Calingaert, and Elizabeth B. Andrews are employees of RTI Health Solutions, a nonprofit research organization, which received funding from Teva Pharmaceutical Industries, Ltd. to conduct this study. The contract between RTI Health Solutions and Teva assured independent publication rights. Sigal Kaplan is an employee of Teva Pharmaceuticals Industries Ltd. Mark Forrest Gordon is an employee of Teva Branded Pharmaceutical Products R&D, Inc.

## ETHICS STATEMENT

This research was approved by the RTI IRB and the CMS privacy board.

## Supporting information


**Appendix** S1: Supporting Information
