# Supplementary material for: The risk of melanoma with rasagiline compared with other antiparkinsonian medications: A retrospective cohort study in the United States medicare database
Source: Pharmacoepidemiol Drug Saf. 2022 Apr 6;31(6):643–51. doi: 10.1002/pds.5422 (PMC9321028; doi:10.1002/pds.5422)
Supplement: Supplementary file 1 — Appendix S1: Supporting Information [file PDS-31-643-s001.docx]

Online Supplement

Table of Contents

[LIST OF TABLES 1](#_Toc74063148)

[LIST OF FIGURES 1](#_Toc74063149)

[1 eMethods 8](#_Toc74063150)

[1.1 Analysis of Additional Cohorts 8](#_Toc74063151)

[1.2 Validation of Melanoma and Nonmelanoma Skin Cancer 14](#_Toc74063152)

[1.3 Quantitative Bias Analysis 20](#_Toc74063153)

[2 References 21](#_Toc74063154)

LIST OF TABLES

[eTable A-1. Antiparkinsonian Drugs Used for Construction of Study Cohorts 2](#_Toc74063155)

[eTable A-2. Description of Potential Confounding Variables Used for Descriptive Analyses and Adjustment 3](#_Toc74063156)

[eTable A-3. Distribution of Index Antiparkinsonian Medication for Patients in the Other Antiparkinsonian Drug Cohort Without Selegiline Use On or Before the Cohort Entry Date (N = 96,552) 5](#_Toc74063157)

[eTable B-1. Baseline Characteristics of Patients in the Parkinson’s Disease Cohort and the Non-Parkinson’s Disease Cohort 10](#_Toc74063158)

[eTable B-2. Health Care Resource Use During the 180 Days Before and Including the Cohort Entry Date, Parkinson’s Disease Cohort and Non-Parkinson’s Disease Cohort 12](#_Toc74063159)

[eTable B-3. Comparison of Melanoma and Nonmelanoma Skin Cancer Between the Parkinson’s Disease Cohort and the Non-Parkinson’s Disease Cohort 13](#_Toc74063160)

[eTable B-4. Melanoma Diagnosis Codes 16](#_Toc74063161)

[eTable B-5. Nonmelanoma Skin Cancer Diagnosis Codes 18](#_Toc74063162)

[eTable B- 6. Validation Summary and Positive Predictive Values 20](#_Toc74063163)

[eTable B-7. Results of Quantitative Bias Analysis 21](#_Toc74063164)

LIST OF FIGURES

[eFigure A-1. Steps in Selection of the Study Cohorts From the 1,165,193 Medicare Beneficiaries in the Source Population^a^ 6](#_Toc74063165)

Part A. Tables and Figure

eTable A-1. Antiparkinsonian Drugs Used for Construction of Study Cohorts

| Drug Class and Name |
| --- |
| Levodopa |
| Carbidopa/levodopa (Parcopa, Sinemet, Sinemet CR, Rytary [extended release]) |
| Carbidopa/levodopa/entacapone (Stalevo) |
| Carbidopa/levodopa enteral suspension (Duopa) |
| Levodopa (Dopar, L-Dopa, Larodopa) |
| Dopamine agonists |
| Pramipexole dihydrochloride (Mirapex, Mirapex ER [extended release]) |
| Ropinirole (Requip, Requip XL [extended release]) |
| Apomorphine hydrochloride (Apokyn) |
| Rotigotine (Neupro) |
| Bromocriptine (Parlodel) |
| Cabergoline |
| Monoamine oxidase type-B (MAO-B) inhibitors |
| Selegiline (Eldepryl, Carbex, Zelapar) |
| Rasagiline (Azilect) |
| Catechol-*O*-methyltransferase (COMT) inhibitors |
| Entacapone (Comtan) |
| Tolcapone (Tasmar)—rarely used |
| Anticholinergics |
| Benztropine mesylate (Cogentin) |
| Trihexyphenidyl HCl (Artane) |
| Biperiden (Akineton) |
| Procyclidine (Kemadrin) |
| Other agents |
| Amantadine (Symmetrel) |
| Rivastigmine tartrate (Exelon) |
| Pergolide mesylate (Permax)^a^ |

CR = controlled release; XR = extended release.

Sources: Parkinson’s Disease Foundation. Prescription medications. 2016. Available at: <https://parkinson.org/Understanding-Parkinsons/Treatment/Prescription-Medications>. Accessed May 2, 2019.

WHO Collaborating Centre for Drug Statistics Methodology. ATC/DDD Index 2016. Available at: <http://www.whocc.no/atc_ddd_index/>. Accessed May 2, 2019.

^a^ Withdrawn from the US market March 29, 2007.

eTable A-2. Description of Potential Confounding Variables Used for Descriptive Analyses and Adjustment

| Variable | Levels |
| --- | --- |
| **Demographic** |  |
| Age at cohort entry, y | 65-69, 70-74, 75-79, 80-84, 85+ |
| Sex | Male, female |
| Race/ethnicity | White, black, Asian, Hispanic, other, unknown |
| Region of residence | Northeast, South, Midwest, West |
| Calendar year of cohort entry | 2006-2015 |
| Low-income subsidy status | Yes, No |
| **Medical comorbidities** |  |
| Charlson Comorbidity Index (CCI)^a^ |  |
| Modified summary Score^b^ | 0, 1-2, 3-4, 5 |
| Individual variables that made up CCI (points each contribute to summary score are in parentheses) |  |
| Myocardial infarction (1) | Yes, No |
| Congestive heart failure (1) | Yes, No |
| Peripheral vascular disease (1) | Yes, No |
| Cerebrovascular disease (1) | Yes, No |
| Chronic pulmonary disease (1) | Yes, No |
| Connective tissue disease (1) | Yes, No |
| Dementia (1) | Yes, No |
| Ulcer disease (1) | Yes, No |
| Mild liver disease (1) | Yes, No |
| Moderate or severe liver disease (3) | Yes, No |
| Diabetes (1) | Yes, No |
| Diabetes with end organ damage (2) | Yes, No |
| Hemiplegia (paralysis) (1) | Yes, No |
| Moderate or severe renal disease (2) | Yes, No |
| Any malignancy, including leukemia and lymphoma (2) | Yes, No |
| Metastatic solid tumor (6) | Yes, No |
| AIDS (6) | Yes, No |
| Other chronic medical conditions |  |
| Asthma | Yes, No |
| Organ transplant | Yes, No |
| Immunosuppressive disorders (other than those listed in the CCI) | Yes, No |
| Hyperlipidemia | Yes, No |
| **Health care resource utilization in the 180 days before and including cohort entry date** |  |
| Primary care physician visits | 0, 1, 2, 3+ |
| Visits to a neurologist or neurosurgeon | 0, 1, 2+ |
| Dermatologist visits | 0, 1, 2+ |
| Hospitalizations for any cause | 0, 1, 2+ |
| Hospitalizations for PD (PD was recorded as the admission diagnosis) | 0, 1, 2+ |
| Number of skin biopsies | 0, 1, 2+ |
| **Comedications** |  |
| Non-antiparkinsonian medications in the 180 days before and including the cohort entry date |  |
| Immunosuppressants including corticosteroids | Yes, No |
| Nonsteroidal anti-inflammatory drugs (NSAIDs) | Yes, No |
| Antihypertensives or diuretics | Yes, No |
| Antirheumatic agents (other than corticosteroids and NSAIDs) | Yes, No |
| Antiparkinsonian medications (other than the index exposure) in the 180 days before cohort entry |  |
| Levodopa, with or without use of carbidopa or a COMT inhibitor such as entacapone | Yes, No |
| Dopamine agonist | Yes, No |
| Anticholinergic | Yes, No |
| Other agents (amantadine, rivastigmine tartrate, and pergolide mesylate) | Yes, No |
| Concurrent use of levodopa or other dopamine agonists at the cohort entry date^c^ | 1) No concurrent use 2) Concurrent use of a single levodopa drug (with or without use of carbidopa or a COMT inhibitor), regardless of use of any other antiparkinsonian drug 3) Concurrent use of a single dopamine agonist drug, regardless of use of any other antiparkinsonian drug 4) Concurrent use of more than 1 levodopa drug (with or without use of carbidopa or a COMT inhibitor) or more than 1 dopamine agonist drug, or any combination of drug(s) in both classes, regardless of use of any other antiparkinsonian drug 5) Use of any other antiparkinsonian drug (anticholinergics and other medications) with no levodopa drug and no dopamine agonist |

COMT = catechol-*O*-methyltransferase; PD = Parkinson’s disease.

^a^ Medical conditions in the CCI were assessed using all available history before or on the cohort entry date using diagnosis codes from inpatient, outpatient, and carrier claims files.

^b^ Modified CCI summary score was calculated using a SAS macro program developed by Cenzer.^1^

^c^ Concurrent use was defined as filling a prescription for levodopa or other dopamine agonist either on the cohort entry date or in both the 30-day period before the cohort entry date and the 30-day period after the cohort entry date.

eTable A-3. Distribution of Index Antiparkinsonian Medication for Patients in the Other Antiparkinsonian Drug Cohort Without Selegiline Use On or Before the Cohort Entry Date (N = 96,552)

| Type of Index Medication | Number | Percentage |
| --- | --- | --- |
| Amantadine | 6,774 | 7.02 |
| Apomorphine hydrochloride | 76 | 0.08 |
| Benztropine mesylate | 2,999 | 3.11 |
| Biperiden | NR | NR |
| Bromocriptine | 239 | 0.25 |
| Cabergoline | 26 | 0.03 |
| Carbidopa/levodopa | 43,630 | 45.19 |
| Carbidopa/levodopa/entacapone | 3,631 | 3.76 |
| Entacapone | 4,230 | 4.38 |
| Pergolide mesylate | 18 | 0.02 |
| Pramipexole dihydrochloride | 9,555 | 9.90 |
| Procyclidine | NR | NR |
| Rivastigmine tartrate | 9,377 | 9.71 |
| Ropinirole | 12,006 | 12.43 |
| Rotigotine | 2,048 | 2.12 |
| Tolcapone | 30 | 0.03 |
| Trihexyphenidyl HCL | 1,900 | 1.97 |

HCL = hydrochloride; NR = not reported because of the CMS cell suppression policy that prohibits the reporting of cell values of 1 to 10.

eFigure A-1. Steps in Selection of the Study Cohorts From the 1,165,193 Medicare Beneficiaries in the Source Population^a^


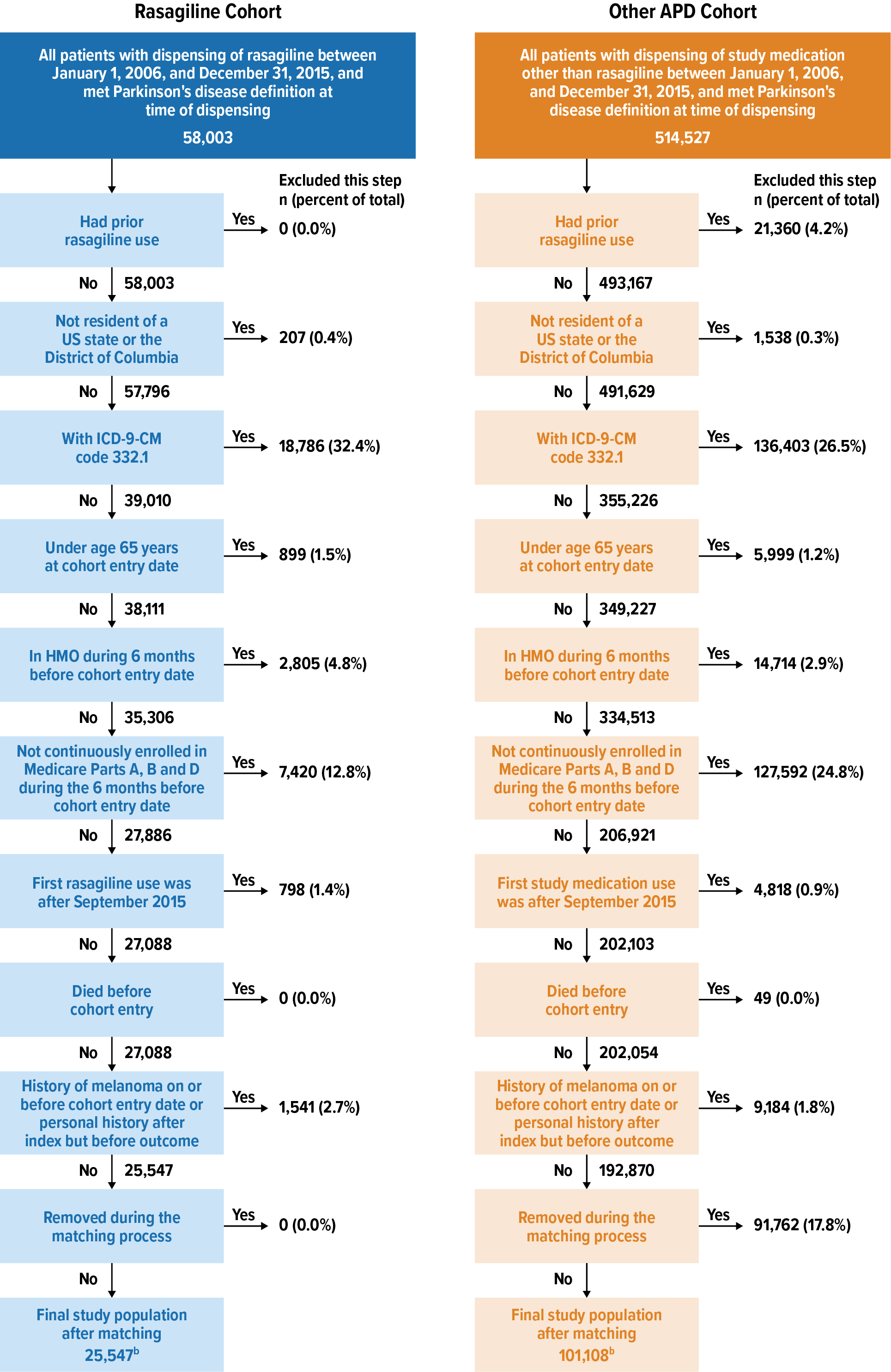


APD = Antiparkinsonian drug other than rasagiline; HMO = health maintenance organization; ICD-9-CM = *International Classification of Diseases, Ninth Revision, Clinical Modification*; US = United States.

^a^Medicare beneficiaries (1) enrolled in fee-for-service Medicare insurance and aged 65 years or older; (2) continuously enrolled in Medicare Parts A, B, and D for at least 6 months during 2006-2015; and (3) with at least 1 inpatient or outpatient claim with an ICD-9-CM diagnosis code of 332.0.

^b^ For the main analysis described in the manuscript, selegiline users were excluded from both cohorts. This resulted in the rasagiline cohort decreasing in size to 23,708 and the APD cohort to 96,552.

Part B. emethods and tables

# eMethods

## Analysis of Additional Cohorts

We performed an analysis in two additional cohorts to compare the incidence of melanoma and nonmelanoma skin cancer (NMSC) between patients with Parkinson’s disease (PD) receiving treatment with non-rasagiline antiparkinsonian drugs (APDs) and patients without PD.

The overall source population comprised adults 65 years of age or older enrolled in fee-for-service Medicare insurance with data in the Medicare Research Identifiable Files. Patients were eligible for possible study inclusion if they had at least 6 months of continuous enrollment in Medicare Parts A, B, and D during the study period, 2006-2015, during which they were not enrolled through a managed care program. This was considered the “eligible source population.”

Data were requested for 2 groups of patients from the eligible source population:

- Patients meeting a broad definition of PD: at least 1 medical claim with a diagnosis code of PD (1 inpatient or outpatient or physician claim with an *International Classification of Diseases, Ninth Revision, Clinical Modification* [ICD‑9‑CM] diagnosis code of 332.0) during the study period; all years of available data were requested dating back through 2006 for the Part D data and through 1999 for all other data files.
- Patients without PD diagnoses from a random sample of the eligible source population that was 5 times the size of the sample that met the broad definition of PD. Patients who did not have any claim with a diagnosis of PD (i.e., no claims for ICD‑9‑CM 332.0 or 332.1 recorded at any time during 2006-2015) were included. For each patient, Medicare data recorded at any time during the study period were requested.

Two cohorts were constructed as follows:

**Non-rasagiline PD cohort**

- Random sample of patients selected from the eligible study population who met the broad definition of PD and were receiving treatment with any non-rasagiline APD during the study period (new or prevalent use of the index medication).
- The cohort entry date was the date of the first dispensed prescription for a non-rasagiline study drug (index medication) during the study period after the following cohort-specific eligibility criteria were met:
- Received a specific non-rasagiline antiparkinsonian study drug during the study period (January 1, 2006, through September 30, 2015). Cohort members were not restricted to new users of APDs.
- Had least 6 months of drug history recorded in the database before the first dispensed prescription of the non-rasagiline APD.
- Met the diagnosis code algorithm definition of PD on or before the cohort entry date. Had at least 2 physician or outpatient claims on separate dates, or at least 2 inpatient claims on separate dates with an ICD‑9‑CM code of 332.0 or *International Classification of Diseases, 10th Revision, Clinical Modification* (ICD-10-CM) code of G20 and no claims with an ICD-9-CM code of 332.1 or ICD-10-CM code of G21.11, G21.19, or G21.8 (secondary parkinsonism: neuroleptic-induced parkinsonism, parkinsonism due to drugs) at any point in time.
- The number of individuals randomly selected to the cohort was the same as that in the cohort of rasagiline new users (of the main study).

**Non-Parkinson’s disease cohort**

- Random sample of patients selected from the eligible study population who did not have a medical history of PD, matched to the non-rasagiline PD cohort on exact age in years, sex, and cohort entry date in a 2 to 1 ratio.
- The cohort entry date for each patient was the cohort entry date of the matched patient in the non-rasagiline PD cohort if the following cohort-specific criteria were met:
- No claim for a physician visit, outpatient visit, or hospitalization coded as ICD‑9‑CM code 332.0 or 332.1 or ICD-10-CM code of G20 or G21.11, G21.19, or G21.8 recorded at any time in the patient’s available history from January 1, 1999, through December 31, 2015.
- No claim for any APD before the cohort entry date. This criterion was implemented to reduce the possibility of including patients with PD who did not have a recorded diagnosis code for PD.

Exclusion criteria:

- Patients who were not residents of a US state or the District of Columbia (i.e., residents of Puerto Rico and other US territories were excluded)
- Patients entitled to Medicare because of end-stage renal disease
- Patients with a history of melanoma on or before the cohort entry date (at least 1 claim with either a diagnosis of malignant melanoma or a personal history of melanoma, ICD‑9‑CM code V10.82)
- Patients with a diagnosis code of personal history of melanoma after the cohort entry date but before having any claim with a melanoma diagnosis code

Each member of each cohort was followed from the cohort entry date to the end of follow-up. The end of follow-up was the earliest of the following events or dates:

- Diagnosis of melanoma (date of first claim for melanoma)
- Disenrollment from Medicare Part A, B, or D, or switch to a managed care plan
- Date of death in Medicare records
- End of study period (December 31, 2015)
- Follow-up in the PD cohort was censored at the date of the first claim for a dispensed prescription of rasagiline
- Follow-up in the non-PD cohort was censored at the date of the first claim with a diagnosis code for PD or at the date of the first dispensed prescription for any APD

Incidence rates for melanoma and NMSC were calculated by dividing the number of events by the total person-time, expressed as the number of events per 100,000 person-years. Events were defined by the same electronic algorithms as were used in the main study for melanoma and NMSC; outcome validation was not performed in this study. Unadjusted and adjusted incidence rate ratios (IRRs) were estimated to compare the incidence of melanoma between the two cohorts. The IRRs were calculated by considering the total person-time of follow-up of each cohort as the person-time at risk. Adjusted IRRs were estimated using Mantel-Haenszel methods for variables other than the matching covariates (age, sex, and calendar year of cohort entry) that were considered important confounders after reviewing the descriptive results. Time-dependent Cox hazards regression models were used to estimate adjusted hazard rates with 95% confidence intervals to compare the hazard rates of melanoma between the PD cohort and the non-PD cohort.

For NMSC, only unadjusted IRRs were calculated.

eTable B-1. Baseline Characteristics of Patients in the Parkinson’s Disease Cohort and the Non-Parkinson’s Disease Cohort

| Characteristic | Parkinson’s Disease Cohort (N = 24,919),  n (%) | Non-Parkinson’s Disease Cohort (N = 51,094),  n (%) |
| --- | --- | --- |
| Sex |  |  |
| Female | 13,353 (53.6) | 27,326 (53.5) |
| Male | 11,566 (46.4) | 23,768 (46.5) |
| Age, y |  |  |
| 65-69 | 3,829 (15.4) | 7,972 (15.6) |
| 70-74 | 4,170 (16.7) | 8,604 (16.8) |
| 75-79 | 5,304 (21.3) | 10,864 (21.3) |
| 80-84 | 5,686 (22.8) | 11,608 (22.7) |
| 85+ | 5,930 (23.8) | 12,046 (23.6) |
| Calendar year of cohort entry |  |  |
| 2006 | 6,550 (26.3) | 13,590 (26.6) |
| 2007 | 2,341 (9.4) | 4,810 (9.4) |
| 2008 | 1,971 (7.9) | 4,036 (7.9) |
| 2009 | 1,886 (7.6) | 3,830 (7.5) |
| 2010 | 1,795 (7.2) | 3,658 (7.2) |
| 2011 | 1,818 (7.3) | 3,708 (7.3) |
| 2012 | 1,991 (8.0) | 4,060 (7.9) |
| 2013 | 2,578 (10.3) | 5,278 (10.3) |
| 2014 | 2,261 (9.1) | 4,618 (9.0) |
| 2015 | 1,728 (6.9) | 3,506 (6.9) |
| Race/ethnicity |  |  |
| White | 21,760 (87.3) | 42,926 (84.0) |
| Black | 1,496 (6.0) | 4,492 (8.8) |
| Asian | 528 (2.1) | 1,287 (2.5) |
| Hispanic | 673 (2.7) | 1,312 (2.6) |
| Other | 380 (1.5) | 877 (1.7) |
| Unknown | 82 (0.3) | 200 (0.4) |
| Geographic region of residence |  |  |
| Midwest | 6,879 (27.6) | 12,814 (25.1) |
| Northeast | 4,450 (17.9) | 10,052 (19.7) |
| South | 9,635 (38.7) | 19,386 (37.9) |
| West | 3,955 (15.9) | 8,842 (17.3) |
| Low-income subsidy status |  |  |
| Yes | 11,087 (44.5) | 16,599 (32.5) |
| No | 13,832 (55.5) | 34,495 (67.5) |
| Comorbidities |  |  |
| CCI comorbidity score^a^ |  |  |
| 0 | 2,676 (10.7) | 10,161 (19.9) |
| 1-2 | 5,961 (23.9) | 15,224 (29.8) |
| 3-4 | 6,096 (24.5) | 11,209 (21.9) |
| 5 or more | 10,186 (40.9) | 14,500 (28.4) |
| History of individual CCI conditions^a^ |  |  |
| Myocardial infarction | 4,097 (16.4) | 7,241 (14.2) |
| Congestive heart failure | 8,819 (35.4) | 13,695 (26.8) |
| Peripheral vascular disease | 10,518 (42.2) | 15,610 (30.6) |
| Cerebrovascular disease | 12,628 (50.7) | 16,171 (31.6) |
| Dementia | 9,396 (37.7) | 6,021 (11.8) |
| Chronic pulmonary disease | 10,761 (43.2) | 19,544 (38.3) |
| Connective tissue disease | 2,080 (8.3) | 3,477 (6.8) |
| Ulcer disease | 2,325 (9.3) | 3,504 (6.9) |
| Mild liver disease | 344 (1.4) | 586 (1.1) |
| Diabetes | 5,564 (22.3) | 10,958 (21.4) |
| Hemiplegia | 1,768 (7.1) | 1,800 (3.5) |
| Moderate or severe renal disease | 5,142 (20.6) | 8,423 (16.5) |
| Diabetes with end organ damage | 5,137 (20.6) | 7,889 (15.4) |
| Any malignancy | 5,151 (20.7) | 11,136 (21.8) |
| Moderate or severe liver disease | 172 (0.7) | 240 (0.5) |
| Metastatic solid tumor | 631 (2.5) | 1,731 (3.4) |
| AIDS | 35 (0.1) | 72 (0.1) |
| History of other medical conditions^a^ |  |  |
| Asthma | 3,514 (14.1) | 6,429 (12.6) |
| Organ transplant | 94 (0.4) | 167 (0.3) |
| Immunosuppressive disorders (other than those listed in the CCI) | 79 (0.3) | 129 (0.3) |
| Hyperlipidemia | 19,129 (76.8) | 39,153 (76.6) |
| Non-antiparkinsonian comedications^b^ |  |  |
| Antihypertensives/diuretics | 15,132 (60.7) | 31,026 (60.7) |
| Antirheumatic agents (other than NSAIDs or corticosteroids) | 192 (0.8) | 450 (0.9) |
| Immunosuppressants including corticosteroids | 2,592 (10.4) | 4,853 (9.5) |
| NSAIDs | 3,587 (14.4) | 6,213 (12.2) |

APD = antiparkinsonian drug; CCI = Charlson Comorbidity Index; NSAID = nonsteroidal anti-inflammatory drug.

^a^ Medical conditions were assessed using all available history before or on the cohort entry date.

^b^ Medications were assessed in the 180 days before or on the cohort entry date.

Note: Post hoc, after creation of both cohorts, 628 patients were excluded from the PD cohort because of selegiline use.

eTable B-2. Health Care Resource Use During the 180 Days Before and Including the Cohort Entry Date, Parkinson’s Disease Cohort and Non-Parkinson’s Disease Cohort

| Variable | Parkinson’s Disease Cohort  (N = 24,919) | Non-Parkinson’s Disease Cohort (N = 51,094) |
| --- | --- | --- |
| Primary care physician visits, n (%) |  |  |
| 0 | 4,324 (17.4) | 13,708 (26.8) |
| 1 | 3,062 (12.3) | 8,372 (16.4) |
| 2 | 3,496 (14.0) | 7,909 (15.5) |
| 3+ | 14,037 (56.3) | 21,105 (41.3) |
| Neurologist/neurosurgeon visits, n (%) |  |  |
| 0 | 12,361 (49.6) | 48,007 (94.0) |
| 1 | 4,036 (16.2) | 1,199 (2.3) |
| 2+ | 8,522 (34.2) | 1,888 (3.7) |
| Hospitalizations for any cause, n (%) |  |  |
| 0 | 17,600 (70.6) | 43,702 (85.5) |
| 1 | 2,475 (9.9) | 3,860 (7.6) |
| 2+ | 4,844 (19.4) | 3,532 (6.9) |
| Dermatologist visits, n (%) |  |  |
| 0 | 22,496 (90.3) | 46,014 (90.1) |
| 1 | 1,293 (5.2) | 2,822 (5.5) |
| 2+ | 1,130 (4.5) | 2,258 (4.4) |
| Skin biopsies, n (%) |  |  |
| 0 | 23,579 (94.6) | 48,195 (94.3) |
| 1 | 1,060 (4.3) | 2,348 (4.6) |
| 2+ | 280 (1.1) | 551 (1.1) |
| **Incidence of nonmelanoma skin cancer per 100,000 person-years (95% CI)** | 1,850 (1,731 - 1,975) | 1,909 (1,829 - 1,992) |

CI = confidence interval.

eTable B-3. Comparison of Melanoma and Nonmelanoma Skin Cancer Between the Parkinson’s Disease Cohort and the Non-Parkinson’s Disease Cohort

| Statistic | Parkinson’s Disease Cohort (N = 24,919) | Non-Parkinson’s Disease Cohort (N = 51,094) |
| --- | --- | --- |
| Number of melanoma events | 110 | 426 |
| Person-time (years) | 65,293 | 147,525 |
| Crude incidence rate per 100,000 person-years (95% CI) | 168.5 (138.5-203.1) | 288.8 (262.0-317.5) |
| Unadjusted IRR (95% CI) | 0.58 (0.47-0.72) | Reference |
| Mantel-Haenszel-adjusted IRR | 0.68 (0.55-0.84) | Reference |
| Cox-adjusted HR (95% CI) | 0.58 (0.45-0.76) | Reference |
| Number of NMSC events | 900 | 2,133 |
| Number of patients contributing person-time | 18,919 | 39,906 |
| Person-time (years) | 48,650 | 111,721 |
| Crude incidence rate per 100,000 person-years (95% CI) | 1,850 (1,731-1,975) | 1,908 (1,829-1.992) |
| Unadjusted IRR (95% CI) | 0.97 (0.90-1.05) | Reference |

CI = confidence interval; IRR = incidence rate ratio; NMSC = nonmelanoma skin cancer

Note: Adjusted IRR and Cox-adjusted HR were not calculated for NMSC.

## Validation of Melanoma and Nonmelanoma Skin Cancer

The claims algorithm to identify potential cases of cutaneous melanoma required documentation of a melanoma diagnosis code on at least one inpatient claim or on at least two institutional outpatient or physician visit claims on different dates. The melanoma diagnosis codes were those starting with 172 for ICD-9-CM, which excludes skin of genital organs (184.0-184.9, 187.1-187.9) and sites other than skin, or those starting with C43 for ICD-10-CM. (eTable B-4). For potential cases meeting the claims algorithm based on having two or more outpatient or physician claims, the date of the first claim fulfilling the definition was considered the preliminary event date.

For NMSC, we used an algorithm based on a published, validated definition derived using payer claims data from a US health system that resulted in a positive predictive value (PPV) of 94.9% (95% confidence interval [CI], 92.5%‑97.4%).^2^ This algorithm required documentation of either an ICD-9-CM starting with 173 or ICD-10-CM diagnosis code starting with C44 and a procedure code for treatment that specifies excision, destruction of a malignant lesion, or use of Mohs micrographic technique (eTable B-5).

The claims algorithms were applied to identify the first potential case of cutaneous melanoma and/or NMSC that met the algorithm definition for each of the patients in the study population. We then created patient profiles consisting of chronological listings of all medical claims using all available data on each potential case of cutaneous melanoma and on a random sample of potential NMSC cases. (Because NMSC was a secondary endpoint and the algorithm identified a large number of potential cases [n=4,597], we sought to conserve resources by requesting medical records for a random sample of 200 potential NMSC cases with the goal of obtaining 100 completed abstractions.) Clinical reviewers blinded to treatment reviewed the claims profiles to select medical encounters for record review. During the profile review, the clinical reviewers determined whether the sequence of claims (medical encounters, procedures, diagnosis codes, and medications) was consistent with an incident melanoma (or NMSC when reviewing the NMSC profiles) and selected the most appropriate medical encounter(s) for which to request medical records. Records from medical encounters that were selected for abstraction were those considered most likely to yield the pathology information required to validate case status and to assess melanoma stage at diagnosis. For each potential case, a primary record and a secondary record (backup record to be abstracted if the primary record was not obtainable) were selected by the reviewer. If it was obvious that an individual’s claims were not consistent with the study definition of melanoma (or NMSC), that individual was classified as a noncase and their records were not requested.

After identifying individuals with the encounter of interest, we provided the Centers for Centers for Medicare and Medicaid Services contractor who controls the Medicare research data with a finder file that contained the encrypted patient-identifying number, the date of the medical encounter to be abstracted, the provider or facility name (obtained by linking the provider number from the Medicare claims data to a publicly available data set), the type of service, and the sex and date of birth of the patient. The contractor then added the patient name and securely transferred the data file directly to the medical record abstraction vendor. Requests for medical records (for both the primary and secondary encounters identified during the profile review) were sent by mail to the designated medical provider or facility. Each request packet included descriptive information about the study and procedures; a copy of the institutional review board approval letter and US Health Insurance Portability and Accountability Act (HIPAA) waiver; and detailed instructions for submission of medical record information to the abstraction vendor. Follow-up calls were made to unresponsive providers. If both records were obtained, only the primary record was abstracted. Medical record abstractions were performed by a third-party vendor using a standard abstraction form developed for the study. The abstractors were not informed of the study objectives and were blinded as to cohort assignment of potential cases. The key information collected was a confirmation of the diagnosis of cutaneous melanoma (or NMSC), the date of diagnosis, and for cutaneous melanoma, whether the abstractor could determine from the medical record the stage of melanoma at diagnosis and whether it was the first occurrence of cutaneous melanoma for the patient. If it was determined not to be a first occurrence and the first occurrence was before cohort entry, the patient was classified a noncase. Abstracted data were captured in a Microsoft Access database, which did not contain patient-identifying information or information on study drug exposure. In addition to completed abstraction forms, supporting documentation from the medical records, such as pathology reports of skin biopsies, were photocopied and provided to the clinical reviewers; any patient-identifying or study drug information was redacted.

The clinical reviewers examined all abstracted information to determine the final classification of cases of melanoma or NMSC and, for confirmed melanoma cases, the stage of melanoma and the diagnosis date. Cases were classified as confirmed cases, possible cases, or noncases. Confirmed cases had a documented pathologic diagnosis of skin melanoma. Noncases had a melanoma with a site of origin other than skin (e.g., ocular), had metastatic melanoma without a known skin primary site, or had a different diagnosis that may have been miscoded (e.g., NMSC or myelodysplastic syndrome) with no mention of melanoma in the records available for review. Possible cases were those for which medical records could not be obtained or those for which there was inadequate information available in the medical record to confirm or disprove case status. For most potential cases, both the primary record and secondary record were requested. If both records were received, only the primary record was used in the abstraction and only one record was counted in the denominator of the proportion of records obtained of those requested.

The PPV of each algorithm was defined as the proportion of true cases (medical record–confirmed cases) among all potential cases identified by the algorithm for which either (1) medical records with sufficiently complete information to determine case status were retrieved and abstracted or (2) they were ruled out as potential cases during the profile review process. In the rare instances that potential cases were ruled out during profile review, medical records were not requested. The PPVs for both the melanoma and NMSC claims algorithms were calculated, and as a sensitivity analysis, the PPVs were also calculated, using as the numerator the number of confirmed and possible cases with the same denominator as described above. Exact 95% CIs were calculated for the PPVs using the Clopper-Pearson method for binomial proportions.^4^

A total of 788 potential cases of cutaneous melanoma were identified by the electronic algorithm, and a random sample of 200 potential cases of NMSC were included in the validation component; profile review was conducted for all these potential cases. The clinical reviewers identified 2 potential melanoma cases as noncases and identified 786 for which medical records were requested. Of these, 2 records (1 primary and 1 secondary) were requested for 736 patients, and 1 record was requested for 50 patients for whom no suitable secondary encounter could be identified; therefore, in total, 1,522 records were requested. Of the 200 NMSC potential cases, 2 records were requested for 183 patients and 1 record was requested for 17 patients (383 records total).

The total number of medical records requested for both potential melanoma and NMSC outcomes was 1,905. Of these, 1,307 medical records were not obtained.. The two most common reasons for not obtaining medical records were lack of a response from the provider (76%) and unavailable record (14%).

Of the 786 potential cases for which a medical record was requested, at least one record was obtained for 419 potential cases (53.3% of all potential cases with records requested). Records for all 419 potential cases were abstracted and reviewed by the clinical reviewers. Of these, the clinical reviewer classified 349 (83%) as confirmed cases of cutaneous melanoma, 58 (14%) as possible cases, and 12 (3%) as noncases. The PPV for the melanoma algorithm, for which only confirmed cases were used, was 82.9% (95% CI, 79.0%-86.4%) (eTable B-6). The PPV was similar in each exposure cohort (rasagiline cohort: 84.1%, 95% CI, 77.2%-89.7%; other APD cohort: 82.2%, 95% CI, 77.2%-86.6%. The PPV increased to 96.7% (95% CI, 94.5%‑98.2%) when possible cases were added to confirmed cases in the numerator.

Of the 200 patients whose profiles were reviewed for the NMSC outcome, record retrieval efforts were stopped when data had been abstracted from the medical records of the first 100 patients. Therefore, an accurate estimate for the percentage of patients with records obtained could not be estimated but at a minimum was 50% (100/200). Of these 100 potential cases reviewed by the clinical reviewers, 83 cases were confirmed (83.0%). The PPV for the NMSC algorithm, using confirmed cases in the numerator was 83.0% (95% CI, 74.2%-89.8%). The PPV increased to 99.0% (95% CI, 94.6%‑100.0%) when possible cases were added to confirmed cases in the numerator (eTable B-6).

eTable B-4. Melanoma Diagnosis Codes

| Code | Description |
| --- | --- |
| **ICD‑9‑CM** |  |
| **172.0** | Malignant melanoma of skin of lip |
| 172.1 | Malignant melanoma of skin of eyelid, including canthus |
| 172.2 | Malignant melanoma of skin of ear and external auditory canal |
| **172.3** | **Malignant melanoma of skin of other and unspecified parts of face** |
| 172.4 | Malignant melanoma of skin of scalp and neck |
| 172.5 | Malignant melanoma of skin of trunk, except scrotum |
| 172.6 | Malignant melanoma of skin of upper limb, including shoulder |
| 172.7 | Malignant melanoma of skin of lower limb, including hip |
| 172.8 | Malignant melanoma of other specified sites of skin |
| 172.9 | Melanoma of skin, site unspecified |
| **ICD‑10‑CM** |  |
| C43 | Malignant melanoma of skin |
| C43.0 | Malignant melanoma of lip |
| C43.1 | Malignant melanoma of eyelid, including canthus |
| C43.10 | Malignant melanoma of unspecified eyelid, including canthus |
| C43.11 | Malignant melanoma of right eyelid, including canthus |
| C43.12 | Malignant melanoma of left eyelid, including canthus |
| C43.2 | Malignant melanoma of ear and external auricular canal |
| C43.20 | Malignant melanoma of unspecified ear and external auricular canal |
| C43.21 | Malignant melanoma of right ear and external auricular canal |
| C43.22 | Malignant melanoma of left ear and external auricular canal |
| C43.3 | Malignant melanoma of other and unspecified parts of face |
| C43.30 | Malignant melanoma of unspecified part of face |
| C43.31 | Malignant melanoma of nose |
| C43.39 | Malignant melanoma of other parts of face |
| C43.4 | Malignant melanoma of scalp and neck |
| C43.5 | Malignant melanoma of trunk |
| C43.51 | Malignant melanoma of anal skin |
| C43.52 | Malignant melanoma of skin of breast |
| C43.59 | Malignant melanoma of other part of trunk |
| C43.6 | Malignant melanoma of upper limb, including shoulder |
| C43.60 | Malignant melanoma of unspecified upper limb, including shoulder |
| C43.61 | Malignant melanoma of right upper limb, including shoulder |
| C43.62 | Malignant melanoma of left upper limb, including shoulder |
| C43.7 | Malignant melanoma of lower limb, including hip |
| C43.70 | Malignant melanoma of unspecified lower limb, including hip |
| C43.71 | Malignant melanoma of right lower limb, including hip |
| C43.72 | Malignant melanoma of left lower limb, including hip |
| C43.8 | Malignant melanoma of overlapping sites of skin |
| C43.9 | Malignant melanoma of skin, unspecified |
| D03.21 | Melanoma in situ of right ear and external auricular canal |
| D03.22 | Melanoma in situ of left ear and external auricular canal |
| D03.3 | Melanoma in situ of other and unspecified parts of face |
| D03.30 | Melanoma in situ of unspecified part of face |
| D03.39 | Melanoma in situ of other parts of face |
| D03.4 | Melanoma in situ of scalp and neck |
| D03.5 | Melanoma in situ of trunk |
| D03.51 | Melanoma in situ of anal skin |
| D03.52 | Melanoma in situ of breast (skin) (soft tissue) |
| D03.59 | Melanoma in situ of other part of trunk |
| D03.6 | Melanoma in situ of upper limb, including shoulder |
| D03.60 | Melanoma in situ of unspecified upper limb, including shoulder |
| D03.61 | Melanoma in situ of right upper limb, including shoulder |
| D03.62 | Melanoma in situ of left upper limb, including shoulder |
| D03.7 | Melanoma in situ of lower limb, including hip |
| D03.70 | Melanoma in situ of unspecified lower limb, including hip |
| D03.71 | Melanoma in situ of right lower limb, including hip |
| **D03.72** | **Melanoma in situ of left lower limb, including hip** |
| D03.8 | Melanoma in situ of other sites |
| D03.9 | Melanoma in situ, unspecified |

Source: <http://www.icd9data.com/>.

ICD‑9‑CM = *International Classification of Diseases, 9th Revision, Clinical Modification*; ICD‑10‑CM = *International Classification of Diseases, 10th Revision, Clinical Modification*.

eTable B-5. Nonmelanoma Skin Cancer Diagnosis Codes

| Code | Description |
| --- | --- |
| **ICD‑9‑CM** |  |
| **173.0** | Other malignant neoplasm of skin of lip |
| 173.1 | Other malignant neoplasm of skin of eyelid including canthus |
| 173.2 | Other malignant neoplasm of skin of ear and external auditory canal |
| **173.3** | **Other malignant neoplasm of skin of other and unspecified parts of face** |
| 173.4 | Other malignant neoplasm of scalp and skin of neck |
| 173.5 | Other malignant neoplasm of skin of trunk except scrotum |
| 173.6 | Other malignant neoplasm of skin of upper limb including shoulder |
| 173.7 | Other malignant neoplasm of skin of lower limb including hip |
| 173.8 | Other malignant neoplasm of other specified sites of skin |
| 173.9 | Other malignant neoplasm of skin site unspecified |
| **ICD‑10‑CM** |  |
| C44.0 | Other and unspecified malignant neoplasm of skin of lip |
| C44.1 | Other and unspecified malignant neoplasm of skin of eyelid, including canthus |
| C44.2 | Other and unspecified malignant neoplasm of skin of ear and external auricular canal |
| C44.3 | Other and unspecified malignant neoplasm of skin of other and unspecified parts of face |
| C44.4 | Other and unspecified malignant neoplasm of skin of scalp and neck |
| C44.5 | Other and unspecified malignant neoplasm of skin of trunk |
| C44.6 | Other and unspecified malignant neoplasm of skin of upper limb, including shoulder |
| C44.7 | Other and unspecified malignant neoplasm of lower limb, including hip |
| C44.8 | Other and unspecified malignant neoplasm of overlapping sites of skin |
| C44.9 | Other and unspecified malignant neoplasm of skin, unspecified |
| **CPT** |  |
| 11600-11606 | Excision, malignant lesion including margins, trunk, arms, or legs (by size, cm) |
| 11600 | Exc tr-ext mal+marg 0.5 cm/< |
| 11601 | Exc tr-ext mal+marg 0.6-1 cm |
| 11602 | Exc tr-ext mal+marg 1.1-2 cm |
| 11603 | Exc tr-ext mal+marg 2.1-3 cm |
| 11604 | Exc tr-ext mal+marg 3.1-4 cm |
| 11606 | Exc tr-ext mal+marg > 4 cm |
| 11620 | Exc h-f-nk-sp mal+marg 0.5/< |
| 11621 | Exc s/n/h/f/g mal+mrg 0.6-1 |
| 11622 | Exc s/n/h/f/g mal+mrg 1.1-2 |
| 11623 | Exc s/n/h/f/g mal+mrg 2.1-3 |
| 11624 | Exc s/n/h/f/g mal+mrg 3.1-4 |
| 11626 | Exc s/n/h/f/g mal+mrg > 4 cm |
| 11640-11646 | Excision, malignant lesion including margins, face, ears, eyelids, nose, lips (by size, cm) |
| 11640 | Exc f/e/e/n/l mal+mrg 0.5 cm< |
| 11641 | Exc f/e/e/n/l mal+mrg 0.6-1 |
| 11642 | Exc f/e/e/n/l mal+mrg 1.1-2 |
| 11643 | Exc f/e/e/n/l mal+mrg 2.1-3 |
| 11644 | Exc f/e/e/n/l mal+mrg 3.1-4 |
| 11646 | Exc f/e/e/n/l mal+mrg > 4 cm |
| 17260-17266 | Destruction, malignant lesion trunk, arms, or legs |
| 17260 | Destruction of skin lesions |
| 17261 | Destruction of skin lesions |
| 17262 | Destruction of skin lesions |
| 17263 | Destruction of skin lesions |
| 17264 | Destruction of skin lesions |
| 17266 | Destruction of skin lesions |
| 17270-17276 | Destruction, malignant lesion, scalp, neck, hands, feet, genitalia |
| 17270 | Destruction of skin lesions |
| 17271 | Destruction of skin lesions |
| 17272 | Destruction of skin lesions |
| 17273 | Destruction of skin lesions |
| 17274 | Destruction of skin lesions |
| 17276 | Destruction of skin lesions |
| 17280-17286 | Destruction, malignant lesion, face, ears, eyelids, nose, lips |
| 17280 | Destruction of skin lesions |
| 17281 | Destruction of skin lesions |
| 17282 | Destruction of skin lesions |
| 17283 | Destruction of skin lesions |
| 17284 | Destruction of skin lesions |
| 17286 | Destruction of skin lesions |
| 17311-17315 | Chemosurgery (Mohs micrographic technique) |
| 17311 | Mohs 1 stage h/n/hf/g |
| 17312 | Mohs addl stage |
| 17313 | Mohs 1 stage t/a/l |
| 17314 | Mohs addl stage t/a/l |
| 17315 | Mohs surg addl block |

Source: <http://www.icd9data.com/>.

CPT = Current Procedural Terminology; ICD‑9‑CM = International Classification of Diseases, 9th Revision, Clinical Modification; ICD‑10‑CM = International Classification of Diseases, 10th Revision, Clinical Modification.

eTable B- 6. Validation Summary and Positive Predictive Values

|  | Melanoma | Nonmelanoma Skin Cancers |
| --- | --- | --- |
| Potential cases identified by algorithm | 788 | 4,597 |
| Potential cases with profiles created | 788 | 200 |
| Potential cases judged noncases during profile review | 2 | 0 |
| Potential cases with record requested | 786 | 200 |
| Potential cases with record not obtained/abstracted^a^ | 367 | 100 |
| Potential cases with record obtained and abstracted | 419 | 100 |
| Confirmed case | 349 | 83 |
| Possible case^b^ | NR | NR |
| Noncase | NR | NR |
| PPV-confirmed cases^c^, (95% CI) | 82.9% (79.0%-86.4%) | 83.0% (74.2%-89.8%) |
| PPV-confirmed and possible cases^d^, (95% CI) | 96.7% (94.5%-98.2%) | 99.0% (94.6%-100.0%) |

CI = confidence interval; NR = NR = not reportable in order to mask small number; PPV = positive predictive value.

Note: According to CMS' cell size suppression policy, any cell containing a frequency value of 1 to 10, or any cell that allows a value of 1 to 10 to be derived from other reported cells or information, cannot be reported under the Medicare data use agreement.

^a^ For potential NMSC cases, record retrieval and abstraction was stopped after records were obtained for 100 potential cases. For potential melanoma cases, data were abstracted for every patient who had records obtained, so this row is a count of those for whom records were not obtained.

^b^ Possible cases are potential cases for whom inadequate information was available in the medical record to confirm the case status.

^c^ The numerator is the number of confirmed cases, and the denominator is the number of medical records obtained and abstracted plus the number of cases determined to be noncases by profile review.

^d^ The numerator is the number of confirmed and possible cases, and the denominator is the number of medical records obtained and abstracted plus the number of cases determined to be noncases by profile review.

## Quantitative Bias Analysis

We performed a post hoc analysis to assess the potential impact of residual confounding on the observed results in our study. We used a method described in Schneeweiss.^3^

The table below shows various combinations of values for the strength of association between a hypothetical binary, unmeasured confounder and cutaneous melanoma (RR_cd_) and the prevalence of the unmeasured confounder in the rasagiline exposed (P_C1_) and unexposed (P_C0_) cohorts that achieve the relative risk we observed (ARR) when the true relative risk is 1.0.

eTable B-7. Results of Quantitative Bias Analysis

| RR | P_C1_ | P_C0_ | RR_cd_ | ARR |
| --- | --- | --- | --- | --- |
| 1.0 | 0.5 | 0.26 | 4.0 | 1.40 |
| 1.0 | 0.4 | 0.19 | 4.0 | 1.40 |
| 1.0 | 0.3 | 0.12 | 4.0 | 1.40 |
| 1.0 | 0.5 | 0.21 | 3.0 | 1.41 |
| 1.0 | 0.4 | 0.14 | 3.0 | 1.41 |
| 1.0 | 0.3 | 0.07 | 3.0 | 1.40 |
| 1.0 | 0.5 | 0.16 | 2.5 | 1.41 |
| 1.0 | 0.4 | 0.10 | 2.5 | 1.39 |
| 1.0 | 0.3 | 0.03 | 2.5 | 1.39 |

RR = true relative risk.

P_C1_ = prevalence of unmeasured confounder in exposed (Cohort A).

P_C0_ = prevalence of unmeasured confounder in unexposed (Cohort B).

RR_cd_ = relative risk of association between confounder and disease.

ARR = apparent relative risk (i.e., the confounded relative risk). (Calculated using formula [1a], p. 294 in Schneeweiss^3^).

From this table it is apparent that to achieve the observed study results if the true relative risk were in fact 1.0 would take a combination of both a strong relationship between the unmeasured confounder and the outcome as well as a large difference in the prevalence of the confounder between the rasagiline exposed and unexposed cohorts.

# References

1. Cenzer IS. Macro computing Charlson Comorbidity Index from CMS claims data. 2013. Accessed August 30, 2019. <https://www.lexjansen.com/wuss/2013/119_Paper.pdf>

2. Eide MJ, Krajenta R, Johnson D, et al. Identification of patients with nonmelanoma skin cancer using health maintenance organization claims data. *Am J Epidemiol*. 2010 Jan 1;171(1):123-128.

3. Schneeweiss S. Sensitivity analysis and external adjustment for unmeasured confounders in epidemiologic database studies of therapeutics. *Pharmacoepidemiol Drug Saf*. 2006 May;15(5):291-303.

4. Clopper C, Pearson ES. The use of confidence or fiducial limits illustrated in the case of the binomial. *Biometrika*. 1934;26:404-413.
